# Consistent Selection towards Low Activity Phenotypes When Catchability Depends on Encounters among Human Predators and Fish

**DOI:** 10.1371/journal.pone.0048030

**Published:** 2012-10-24

**Authors:** Josep Alós, Miquel Palmer, Robert Arlinghaus

**Affiliations:** 1 Instituto Mediterráneo de Estudios Avanzados, IMEDEA (CSIC-UIB), Esporles, Illes Balears, Spain; 2 Department of Biology and Ecology of Fishes, Leibniz-Institute of Freshwater Ecology and Inland Fisheries, Berlin, Germany; 3 Inland Fisheries Management Laboratory, Faculty of Agriculture and Horticulture, Humboldt-Universität zu Berlin, Berlin, Germany; Institut Pluridisciplinaire Hubert Curien, France

## Abstract

Together with life-history and underlying physiology, the behavioural variability among fish is one of the three main trait axes that determines the vulnerability to fishing. However, there are only a few studies that have systematically investigated the strength and direction of selection acting on behavioural traits. Using *in situ* fish behaviour revealed by telemetry techniques as input, we developed an individual-based model (IBM) that simulated the Lagrangian trajectory of prey (fish) moving within a confined home range (HR). Fishers exhibiting various prototypical fishing styles targeted these fish in the model. We initially hypothesised that more active and more explorative individuals would be systematically removed under all fished conditions, in turn creating negative selection differentials on low activity phenotypes and maybe on small HR. Our results partly supported these general predictions. Standardised selection differentials were, on average, more negative on HR than on activity. However, in many simulation runs, positive selection pressures on HR were also identified, which resulted from the stochastic properties of the fishes’ movement and its interaction with the human predator. In contrast, there was a consistent negative selection on activity under all types of fishing styles. Therefore, in situations where catchability depends on spatial encounters between human predators and fish, we would predict a consistent selection towards low activity phenotypes and have less faith in the direction of the selection on HR size. Our study is the first theoretical investigation on the direction of fishery-induced selection of behaviour using passive fishing gears. The few empirical studies where catchability of fish was measured in relation to passive fishing techniques, such as gill-nets, traps or recreational fishing, support our predictions that fish in highly exploited situations are, on average, characterised by low swimming activity, stemming, in part, from negative selection on swimming activity.

## Introduction

For decades, humans have selectively removed certain phenotypes and underlying genotypes from wild animal populations [1], [2]. The evolutionary consequences of such harvest have been examined because of the potential risk to sustainable fisheries [3]–[5]. Many genotypic and phenotypic changes in a variety of production-related traits, such as altered reproductive investment, growth rate or size and age at maturation, have been revealed by time series analysis of the phenotypic data for wild populations, many of which were attributed to the intensity and the selective nature of fishing (e.g. [6]–[9]). Cause-and-effect evidence for these changes stems from both experimental evolution in the laboratory (e.g. [10]) and theoretical models (e.g. [11], [12]). Most work on selection by commercial fisheries has focused on life-history evolution [13], whereas the few studies reporting directional selection on fish physiological traits or behavioural phenotypes were completed in a fishing context using passive fishing gear, such as recreational angling, trapping and gill-netting (e.g. [14]–[16]). In such gear, selection on behavioural traits rather than body size might be prevalent due to the passive nature of the gear, which depend on the active decision of a fish to bite on a hook or otherwise encounter the gear [17].

Vulnerability to capture by different fishing gears has been shown to have a genetic component [18], [19], which is an important precondition for fishery-induced selection to result in evolutionary (i.e. genetic) changes rather than mere phenotypic change. Vulnerability to capture is a complex phenotype that includes a range of potentially correlated life-history, physiological, behavioural and morphological traits [17]. Because vulnerability to capture is affected by behavioural decisions of the fish in many fishing gears [20], [21], fishing-induced evolution of behaviour, including activity, home range size and habitat choice, could be prevalent in heavily exploited fish populations [5], [22]–[24]. This is because the vulnerability to either active (e.g. trawling, seining) or passive (e.g. fish traps, long-lining, hook-and-line recreational angling) fishing gear is defined for many individuals by behavioural patterns related to energy acquisition, foraging or refuge-seeking behaviours [25]. However, such behavioural evolution is rarely recognised or studied, in part due to the technological challenges in tracking exploited fish in the wild over large geographical scales.

Theoretically, the odds of catching fish with angling gear and other passive fishing gear should increase with the activity of individual fish (e.g. in gill nets [21], [22], and in long-lining [20]) because with all other factors being equal, activity should increase the likelihood of encountering the gear [22], [26]. However, some fishers, such as recreational anglers, are often also active to some degree, e.g., in spin fishing or when moving around a fishing area by boat. Therefore, the encounter probability of a fish with the gear will also depend on the movement rate of the fisher, but if all factors are considered equally, a more mobile fish should still have a higher chance of encountering the human predator than a less mobile individual [27]. There is conflicting empirical information available in relation to this hypothesis. In a pond-based study Binder et al. [24] failed to find evidence that more vulnerable largemouth bass (*Micropterus salmoides*) genotypes exhibit greater activity compared to less vulnerable individuals. In contrast, Olsen et al. [23] reported that in Atlantic cod (*Gadus morhua*), fish exhibiting greater rates of diel vertical migration were more vulnerable to a range of passive fishing gear but failed to find a relationship between activity space used by the fish and capture probability. More studies on the direction of fishery-induced selection on behavioural traits across a range of fishing styles are needed to derive robust predictions about which direction of behavioural-directed selection to expect in passively fished fisheries.

Most studies on the relationship between behaviour and vulnerability to fishing have inferred behavioural characteristics about individuals under laboratory or other experimental conditions (e.g. [28]) or have assumed a specific behaviour without actually testing it during a catching experiment (e.g. [21]). With the tools of biotelemetry, fish behaviour can be assessed in the wild, and the fish can then be exposed to experimental fishing to quantify vulnerability [29]. The first of such studies have appeared testing the relationship between capture probability and telemetry-based behaviour in the wild [23], [30], [31]. Linking *in situ* spatial behaviour of fish to exploitation promises important new insights into which behavioural traits are likely to be under selection by fishing [23], [32]. It is important to understand whether there are consistent selection pressures across various styles of fishing to more mechanistically and robustly understand the direction of change expected under heavy fishing mortality.

Several authors have previously suggested linking animal movement and fishery-induced evolution in the context of marine fisheries [23], [33], [34]. Such analyses also need to consider the behavioural patterns of fishers, especially in the context of boat-based recreational fisheries or when investigating the fishing patterns at the edges of non-fishing areas (e.g. in the boundaries of marine protected areas) or in the shore-based fishing conditions of lakes, rivers or coastal zones. The selective pressures on activity, exploration or boldness-related behavioural traits across fishing styles appear to remain largely unexplored in situations where both fish and fishers are free to move. One would assume that more mobile fish are particularly likely to be captured with passive fishing gear, but many fisheries (especially recreational fisheries) can be fished passively (by gear that is fixed in a position) but also actively by searching for the target species (e.g. [35]). We nevertheless hypothesise that, independent of fishing style, recreational fishing should select for highly explorative and active individuals that will likely also be more bold and have a greater home range size [36]. We conducted a modelling-based simulation study of realistically moving fish exploited by varying fishing styles. Our objective was to make general predictions about which direction of selection to expect on exploration and activity-related behaviour from recreational fisheries, applied to coastal areas where small-bodied fish with confined home ranges are the usual targets of recreational anglers [37]–[39].

## Materials and Methods

We developed a spatial-explicit individual-based model (IBM) simulation in which prey (fish) and predator (recreational anglers) were simultaneously moving in a range of realistic fishing style scenarios. Individual fish moved following empirically measured ranges of variability patterns of home range (HR) establishment and exploration made by small small-bodied coastal fishes in the Mediterranean Sea (see below). Recreational anglers were assumed to move following four different general behaviours that simulated an increasing degree of spatial complexity from stationary to fully mobile fishing. The approach we took was Lagrangian-based [40], and the trajectory of the fishes and fishers were tracked in the model to determine the encounters and to determine the harvested individuals and the resulting selection pressures on underlying fish behavioural traits.

### Fish Movement Characteristics

**Figure 1:**
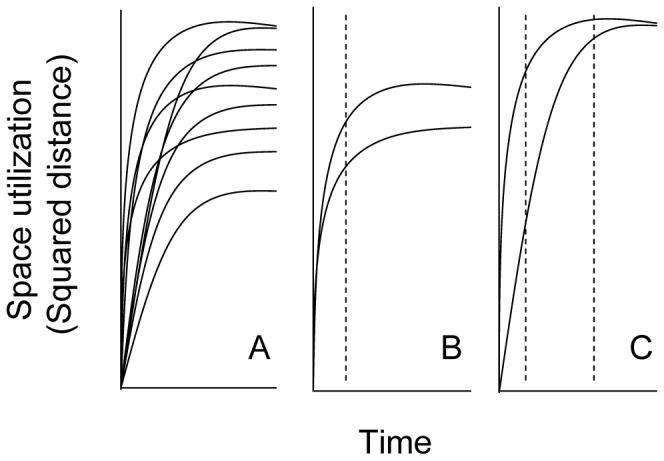
Schematic daily squared displacement over time of a fish moving with a home range (HR). Schematic representation derived from empirical observations using acoustic telemetry techniques in the marine small-bodied fishes *Serranus scriba*, *Serranus cabrilla* and *Xyrichthys novacula* [37], [38], [43]. In all cases, the squared distances reached an asymptote as a characteristic of the general mechanism of the HR behaviour [42]. Two general patterns can be derived from the within-population variability observed: there were some individuals that reached an asymptote at the same time (denoted by the vertical dashed bar) but had different HR sizes (approximated by the ratio *ε/k*, see methods section, panel B), and other individuals with the same HR size (denoted by the vertical dashed bar) but with a different way of exploring the whole HR (approximated by the parameter *k*, see methods section, panel C).

Most of the marine costal fishes move within a well defined HR [41]. When a fish moves following a random walk (RW) the amount of space used increases monotonically with time [42]. Conversely, when a fish moves following a HR-type movement, the amount of space reaches an asymptote because the movement is constrained by an additional rule that links the fish to a specific location or focal point [42]. The characteristics of HR-type movement vary among individuals and may constitute a trait under selection if partly genetically determined as one would generally assume to be the case for most phenotypes, including behavioural phenotypes (e.g. [23], [30], [34], [37], [38], [43] and Figure 1).

HR-behaviour can be mathematically described in different ways [42], one of the best fits is using a biased random walk (BRW), described by an *Ornstein–Uhlenbeck* process [44]. The rationale behind this model is that fish move within a homogeneous environment following random stimuli, but with an additional rule that determines a tendency to remain around a specific point (designated the centre of the HR, [39]). Here we consider that the trajectory of a fish, *r(t) = (x(t), y(t)),* where *x* and *y* are the geographic coordinates at time step *t*, is described by the stochastic *Langevin* equation [45], which yields a *Ornstein-Uhlenbeck* process:
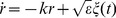
(1)


In this context, the fish moves and is attracted towards the centre of its HR by a central harmonic force of constant *k*, while it is also subjected to a random force described by a Langevin term 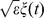, which is a bi-dimensional, white Gaussian process of zero mean, variance *ε* in each spatial coordinate and no correlation among them.

The solution of the equation 1 is:
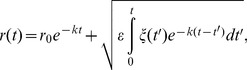
(2)where *r_0_* is the initial position of the fish.

The numerical approximation to the real trajectory sampled with a finite time step *Δt* is given by:
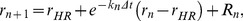
(3)where *r_n_* denotes the position of the fish at time *t_n_ = nΔt*, *r_HR_* is the position of the centre of the HR and *R_n_* is an stochastic, normally distributed term with zero mean and standard deviation approximated by:



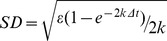
(4)The biological interpretation and implementation of this specific version of BRW for marine coastal fishes is fully developed in Palmer et al. [39]. In our approach, the movement characteristics of HR-type movement can be related to specific parameters in equation 3 as: (1) the size of the circular HR (radius) depends on the ratio *ε/k*, and (2) the exploration rate is related to the parameter *k*, which determines the slope of the curve describing the cumulative space used in function of time. Thus, *k* represents the degree and speed by which an individual moves through its HR. These were the two mathematical descriptors used here to explore fisheries-induced changes on movement characteristics, and were thus considered adaptive traits that very among individuals.

### Fisher Behavioural Characteristics

**Figure 2:**
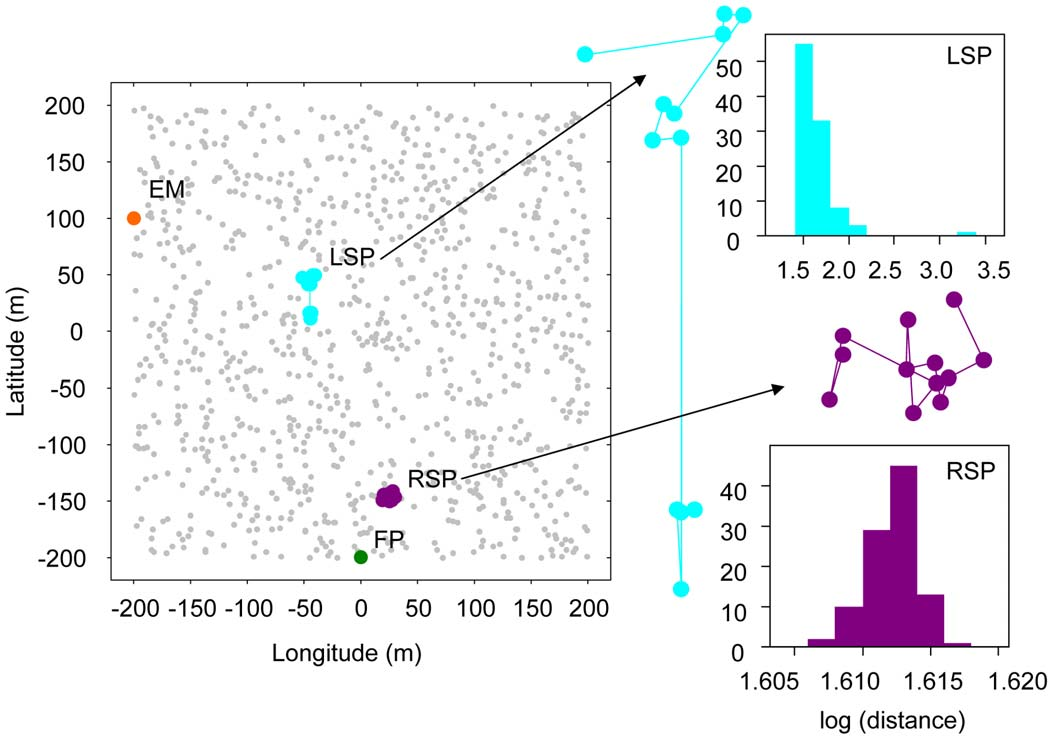
Spatial scenario considered for the IBM simulations. Grey points represent the centre of the HRs of the 1,000 simulated fishes following the biased-random walk described in the methods section. The path (individual Lagrangian trajectory) for one fishing trip (12 time steps) of the fishers following the behaviours described in the M&M are shown: the fisher fixed in one spot (FP), the fisher moving randomly along the edge of the scenario (EM), the random search pattern (RSP) and the Lévy search pattern (LSP). A magnification of the RSP and LSP (at the same spatial scale) is provided to improve visualisation and to show the distribution of the distance travelled between two consecutive time steps during one simulation, presented on a log-scale (m).

We simulated four general human predatory fishing behaviours with an increasing degree of spatial complexity. Scenarios were defined to correspond to typical fishing styles observed in real fisheries by the author team, *viz*.: 1) to fish from a fixed position (FP) similar to, for example, a single access point on a lake, 2) to fish from a random position located at the edge (EM), which equates to random choice of fishing locating along the shoreline of a lake or at the edge of a marine protected area (“fishing along the boundary”), 3) to fish along the activity space of the fish following a random walk path (RSP), and 4) to fish within the activity space of the fish following a Lévy search pattern (LSP) as described previously for real fishing behaviours ([35], Figure 2).

In the FP, the fisher remained both within- and between-fishing trips at the same spot (Figure 2, Table 1). In the EM, the fisher remained at the same place within each fishing trip. However, if at the end of a fishing trip the catch rates were low (approximated by a daily bag <5 fish), the fisher randomly selected another spot at the same shoreline edge for the next fishing trip (Table 1). Many studies have shown that fishers shift among fishing sites depending on catch rates (e.g. [46]), although there is clearly variance in catch-rate dependent fishing site among fisher types [47], that for simplicity was not accounted for in the present paper.

**Table 1:** Characteristics of the movement within (15 min time steps) and between fishing trips in the four fisher behaviours: the fisher fixed in one spot (FP), the fisher moving in the edge of the scenario (EM), the random search pattern (RSP) and the Lévy search pattern (LSP). The daily bag was used as a proxy of the fishing spot quality and as a criterion for changing the fishing spot of the next fishing trip.

	Within fishing trip	Between fishing trip	
		Daily bag ≥5 fishes	Daily bag <5 fishes
FP	Static	Fixed (one spot)	Fixed (one spot)
EM	Static	Fishing spot of the previous day	Random constrained to the edge
RSP	Random walk	Fishing spot of the previous day	Random
LSP	Lévy walk	Fishing spot of the previous day	Random

The third and fourth fisher behaviours included not only the possibility of changing the fishing spot between fishing trips (following the same bag size rule described above for EM), but now the new fishing spot was randomly selected at any site and was not constrained to be localized at the edge as would be possible in boat fishing (Figure 2, Table 1). In addition, fishers were allowed to move across fishing space within a given fishing trip. In the third fisher behaviour scenario (RSM), the fisher moved continuously following a Brownian random walk (the distance travelled per time step was sampled from a normal probability distribution) until the fishing trip finished (Figure 2). The numerical approximation to the fisher’s trajectory with a finite time step *Δt* was given by:
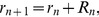
(5)where *r_n_* denotes the position of the fisher at time *t_n_ = nΔt*, and *R_n_* is an stochastic, normally distributed term with zero mean and standard deviation *σ*. A value of *σ* = 1 was selected to obtain trajectories in which the within-trip displacement was, on average, smaller than the between-trip displacement.

In the fourth and final fishing scenario (LSP), the fisher also moved within a fishing trip following a random walk. However, in that case the distance travelled was sampled form heavily skewed probability distribution, following a so called Lévy walk movement ([48], Figure 2). Theoretically, in situations where (human and non-human) animals possess limited information on the distribution of prey, a specialized random walk known as a Lévy flight can yield encounters with sparsely and randomly distributed preys more efficiently than random walks [49]. Indeed, recent evidence suggest that fishers can use search strategies similar to Lévy walks, which conforms to the same search statistics as non-human search strategies (e.g. [35]). Accordingly, the numerical approximation of the fisher’s trajectory with a finite time step *Δt* was given by the same equation 5, but in this case, the stochastic term *R_n_* was not normally distributed but followed a Pareto distribution [50]. The parameters of the Pareto distribution (*L_min_* = 0.1 and *μ* = 1.2) were selected to obtain a mean displacement between consecutive time steps similar to the one obtained when the fisher moved following a Brownian random walk (note, however, that the variance in the case of the Lévy walks is much higher and characterized by rare, long step distances). Random samples from the Pareto distribution were obtained using the function *rpareto* from the *VGAM* library from the R package (V. 2.13.0, http://www.r-project.org/).

### Individual-based Model (IBM) and Simulation Procedures

We developed a spatially-explicit IBM using realistic among-individual fish behavioural variability on movement observed in field trough tracking-observations on small-bodied species most frequently harvested species in the recreational fishery of the Mediterranean (schematized in Figure 1). A set of random movement characteristics were assigned to 1,000 fishes following a realistic normal distribution for the size of the HR (values of *ε/k*: mean 200 m and s.d. 20 m) and a (gamma) distribution for the exploratory/activity-related behaviour (values of *k*: mean 0.1 min^−1^ s.d. 0.2 min^−1^) in the line of this empirical studies [39]. Within-population variability in behaviour was simulated at a time-step of a minute assuming a random distribution as relates to the centre of the HR, always within a realistic scenario of a 400 m×400 m grid due to the overall limited dispersal of our study species [39].

Fish were then exploited in the model by the mentioned four different fishing styles, one style at a time. We assumed a mean fishing trip duration of 3 h in line with empirical estimates of the recreational fishery of this small-sized species [51]. In all fishing styles with dynamic location choice by fishers, the fisher’s location was altered every 15 minutes (Table 1). For simplicity and as a proof-of-concept, we simulated only 1 fisher per scenario. We considered a fish harvested when the fish and the fisher were within 20 m of each other. All the fish harvested were accumulated in the daily fishing bag, which was then used to determine the next day location choice depending on the fishing style scenario. Simulations finished when either 1) only 10% of the 1000 initial fish (i.e. 100 fish, 10% is often considered a collapsed fishery) remained alive, or 2) when no fish was harvested for five consecutive fishing trips. Note that there was no reproduction involved in our simulation, and the final time units emerging until “collapse” are bound to be affected by the assumptions made about catchability and parameters chosen; the absolute results have no real bearing to any real fishery and only serve here as a relative proof-of-concept. Relative results (across fishing styles) are of course valid.

Each fishing style-specific IBM was run 100 times (iterations) serving as parametric bootstrap replicates. We calculated the magnitude of the selection differential (*S*) of each iteration as the difference in the average of the movement traits of surviving fish in the fished (i.e. new parental fish after fishing) and the average movement trait in the original population prior to fishing (ratio *ε/k* and *k*). Because the estimated *Ss* for each movement trait were in different units and exhibited trait-specific variances and means (ratio *ε/k* in m and *k* in min^−1^), *S* values were mean-standardized to evaluate selection strength following Matsumura et al. [52]. To that end, we calculated the mean-standardized selection differential (*Sμ)* as follows:
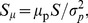
(6)where *S* is the selection differential, *µ_p_* and *σ_p_^2^* are the mean and the variance of the movement trait of the original population prior to fishing. The mean *S_μ_* of the fishing style’s distribution of *S_μ_* (*n* = 100 iterations) was compared between fishing styles and for each of the two movement traits using one-way ANOVA. Normality was tested and the raw data was transformed using Box-Cox transformation. The probability of no selection (*S_μ_* = 0) or positive selection (*S_μ_* >0) was also estimated for each fishing style and movement trait to analyse the consistency of the selection differential across all iterations by re-sampling the distributions of *Sμ* through parametric bootstrap. Modelling and data analysis were completed using the R package.

## Results

**Figure 3:**
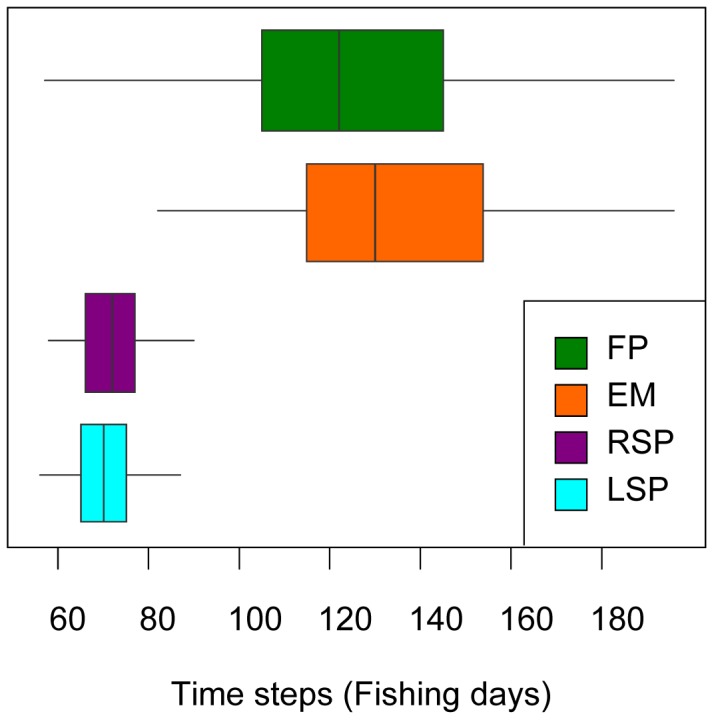
Harvesting efficiency (approximated by the fishing-trips needed to finish the simulation) related to the fisher behaviour. Box-plots of the harvesting efficiencies within 100 simulation runs until the virtual fish population collapsed to <10% of the initial size: one fixed spot (FP, green), edge-based random movement (EM, orange), random search pattern (RSP, purple) and Lévy search pattern (LSP, turquoise). The figure shows the box-plots of the days needed to overexploit the fish stock for each fisher style as a proxy of harvesting efficiency.

The 100 model iterations for each fishing style finished at different time steps (Figure 3). Accordingly, the “shore” or “edge”-bound fisher styles (FP and EM) were less efficient than the boat-based fishing styles in terms of harvesting (fishing duration of FP: 125.3±28.7 days and EM: 134.1±27.1 days, mean and s.d., Figure 3). By contrast, the average fishing days corresponding to exploitation-until-collapse of the random search pattern (RSP: 71.9±7.3 days) and the Lévy search pattern (LSP: 70.5±7.3 days) were shorter indicating more efficient and more rapid harvesting of fish by such type of fishing. There was also less variability within each fishing style compared to fixed- or edge-bound fishing (Figure 3).

**Figure 4:**
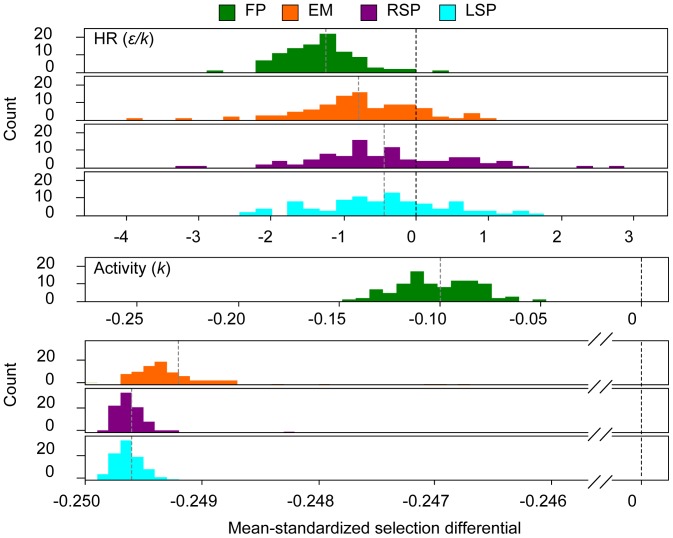
Distribution of mean-standardised selection differentials on the mathematical descriptors of movement. Frequency distributions (*n* = 100) of the mean-standardised selection differentials (*S_μ_*) calculated in each iteration for each movement characteristic (radius of the HR and the activity approximated by *K*) in the different fisher styles (left panels). Black dashed vertical lines mark the level of no selection (*S_μ_* = 0), and grey dashed vertical lines mark the mean-*S_μ_* calculated for the 100 iterations performed for the different fisher styles: the fisher fixed in one spot (FP), the fisher moving in the edge of the scenario (EM), the random search pattern (RSP) and the Lévy search pattern (LSP).

The distribution of the mean-standardized selection differentials (*S_μ_*) for the HR size (ratio *ε/k*) was biased towards negative values (Figure 4), but only in the case of the FP fishing style the mean *S_μ_* was significantly different from 0 or positive values (p = 0.01). In the other three fishing styles, *S_μ_* distributions were not significantly different from zero selection, and positive *S_μ_* commonly occurred (Figure 4). The one-way ANOVA showed statistically differences in the mean *S_μ_* among groups. Accordingly, FP differed from the other three fishing styles (p<0.001 in all cases) obtaining stronger selection differentials on HR size. Similarly, the EM fishing style differed from the other three groups (EM-FP p<0.001; EM RSP<0.05; EM-LSP p<0.05), but differences among the RSP and LSP were not significant (p = 0.99).

The strength of *S_μ_* for the activity-based behaviour was smaller than selection on HR in all cases (Figure 4). However, *S_μ_* were always negative, indicating selective advantages of the low-activity individuals (i.e. those with large *K* values). In this movement trait, the probability of *S_μ_* to be 0 or positive was zero in all fishing styles (Figure 4). The distribution of *S_μ_* values obtained for the FP scenario was again very different from the other three fishing styles, and negative selection differentials on *K* were much more pronounced in edge-based fishing as well as the boat-based fishing styles compared to fixed position fishing (Figure 4) (Post-Hoc test of the one-factorial ANOVA p<0.001 in all two-paired comparisons).

**Figure 5:**
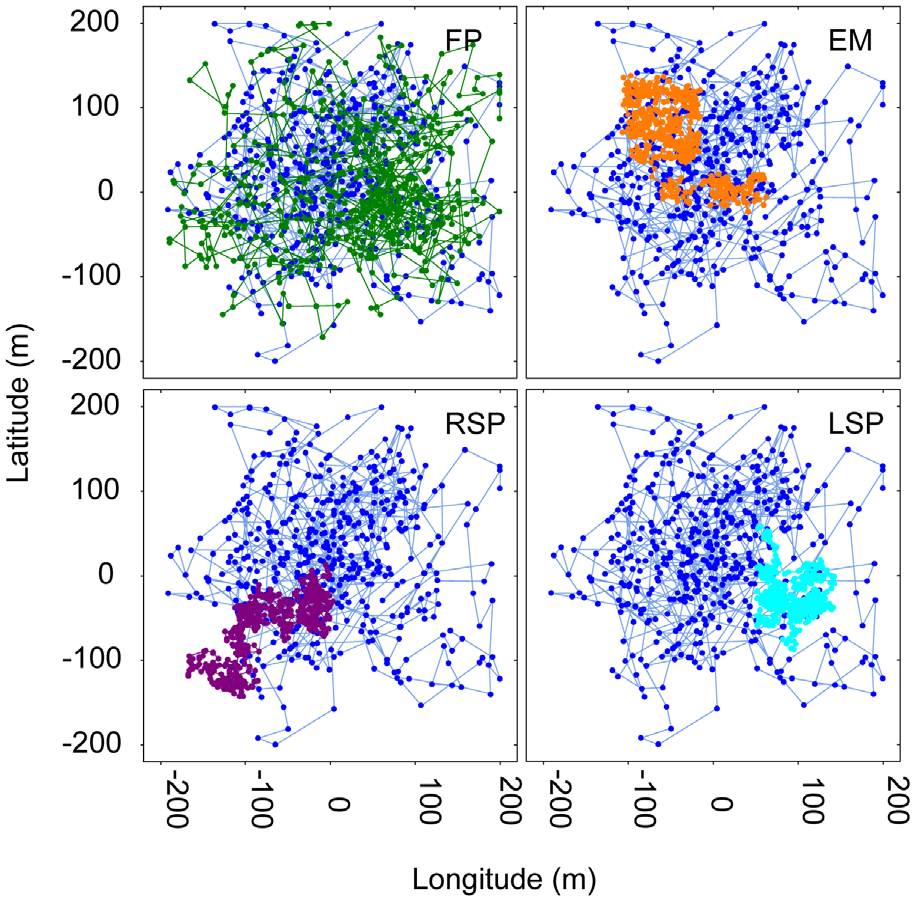
Lagrangian trajectories of the population-average fish in the original and exploited populations. The path of the first 500 time steps of an individual with average movement characteristics (ratio *ε/k* and *k*) sampled from the un-exploited population (in blue) and from the exploited population by the different fisher styles: the fisher fixed in one spot (FP), the fisher moving in the edge of the scenario (EM), the random search pattern (RSP) and Lévy search pattern (LSP). Note the change in the tortuousity degree and a needing for more time steps to reach an asymptote (HR) in the spatial-utilisation in the exploited average fish.

The result of fishing-induced selection on both movement characteristics strongly altered the movement features of the average surviving individual in the exploited population (Figure 5). Surviving individuals were characterized by lower coverage of the whole HR per time, i.e., lower activity (Figure 5). According to our results, such selection should be most pronounced in more active, edge- and boat-based fishing styles (Figure 5).

## Discussion

Fishery-induced evolution (FIE) of behavioural traits constitutes an overlooked component of the emerging concept of evolutionary changes in heritable traits induced by human harvest of fish populations [5], [9], [17], [34], [53]. Although the link between behaviour and the selective nature of a range of fishing gears is often emphasised (e.g. [1], [20], [21], [26]), most studies emphasising the idea of fishing-induced behavioural evolution have used an experimental approach using genotypes selected for high or low vulnerability to capture (e.g. [18], [21]). In our theoretical study, we have investigated selection differentials on two different behavioural traits related to movement features that vary among fish as revealed under natural conditions. The novelty of our research is that the simulated movement patterns emerged from tracking-based assessments of individual variability in behaviour in the wild and that we tested a range of fishing styles likely to happen in nature. Therefore, we could test for the consistency of the selection differential on the two behavioural traits under various realistic fishing styles and therefore produce general results.

To the best of our knowledge, only one study has examined individual vulnerability to capture using telemetry-tracking data collected in the wild in fish. That study in Atlantic cod has provided strong evidence that fishing selects for certain behavioural traits related to diel vertical migration and the use of shallow water areas, both of which increased vulnerability to capture [23]. In line with our study, Olsen et al. [23] did not find a strong selection pressure acting on the use of activity space in Atlantic cod, but fishing gears used in their work were spaced across the activity space and were not fixed at single locations at the edge of the HR. Only in the latter case (fishing from a fixed position) would we predict a very consistent negative selection pressure on HR size (or activity space as a surrogate measure). Overall, however, proving the frequent and intuitive claim that fishing exerts selection pressures on specific behavioural traits has remained elusive and focused on very few species [17], [23], [34], [53]; our work is insightful because it attempts to provide generalisable insights using a parameterisation to small coastal fish species. Naturally, our predictions are contingent on the behaviour of our model species and the fishing styles examined.

A key finding of our results is that all fishing styles exerted consistent negative selection on activity-like behaviour, and at least for the least efficient fishing style of all examined in our work (i.e., fixed spot fishing), there was also a significantly negative selection against individuals with larger HR. Notably, selection differentials on HR were usually larger than those on activity, but they were much less consistent. Moreover, the selection differential on activity behaviour was found to be similarly strong among the more active fishing styles, independent of whether the fishers were moving along the edge or free to move across space. The resulting message of these findings is twofold. First, although the strength of fishery-induced selection has been found to be larger with increasing fishing mortality (e.g. [13]), it is the interplay of fish and fisher behaviour and the particular overlap of the two in space that determines the selection pressures that yield the ultimate result, at least in terms of behavioural traits and likely also in terms of other correlated life-history traits [23]. However, most previous work on fishery-induced selection has only focused on the selective nature of the fishing gear from a fish perspective (e.g. with respect to size selectivity, [4]). Therefore, fishery-induced selection pressures that arise from choosing a fishing site in space are underestimated, and recommendations by Mehner et al. [54] for example, to counter fisheries-induced selection pressures by marine protected areas may not hold in reality. Future models should thus consider the importance of the type of fishing operations and the overlap of geometries between fish and fishers when attempting to study selection on behavioural traits.

The second key message of our paper is that our simulation-based results underscored the generic observations made that passive fishing gear, such as long-lining or gill nets, should consistently select against more active individuals [20]–[22], [26]. Although theoretical models demonstrating the importance of movement strategy in determining the encounter rates with prey are well described (e.g. [27]), studies in line with our model findings and empirical studies evaluating the exact relationship with the vulnerability to capture in fish are still scarce. Using passive integrated transponders, Klefoth et al. [55] found that more active carp (*Cyprinus carpio*) individuals were more vulnerable to angling, which is a finding similar to our model. Similarly, Biro & Post [21] found that more active rainbow trout (*Oncorhynchus mykiss*) were more likely fall victim to gill nets compared to less active fish, and Olsen et al. [23] demonstrated that Atlantic cod performing more intensive vertical movements were more vulnerable to a range of passive fishing gears. By contrast, largemouth bass (*Micropterus salmoides*) selected for high vulnerability to recreational angling did not exhibit greater locomotory activity than low vulnerability genotypes in ponds [24], and in that case, aggression rather than activity per se seems to be more important for catchability [14]. However, it is uncertain whether this result holds for more extended lakes and rivers where individuals can better express their behavioural patterns and where experimental scale and edge effect are likely to matter less. Therefore, a key prediction emerging from our work is that in all cases where the encounter between a fish and a gear determines a fishes’ vulnerability and under the assumption that fishers exploit a large component of the HR of a particular fish (e.g. by boat fishing a lake or the entire HR of a small coastal fish), one would expect consistent selection towards low activity behavioural phenotypes and occasional very strong selection for low HR size. This is a suitable null hypothesis for all future work on fishery-induced selection of activity-based and activity-space behaviours in the many species where aggression is not primarily responsible for catchability. Obviously, our results will depend on the habitat features in place (e.g. availability of save refuges) and the co-variance of activity with habitat choice. This is an open question for the future.

Our study focused on movement-based behavioural phenotypes, omitting other behaviours potentially under selection in fishing. Indeed, in Atlantic cod, genotypes preferring near shore areas were found to be selectively removed over time, and therefore, habitat choice might be a trait under selection in addition to activity [23], [53]. Moreover, there is an emerging literature on ecologically relevant personality traits in fish [56]. Although in many cases, major personality or temperament traits such as exploration, activity, boldness and aggression [36] are correlated across contexts to form behavioural syndromes (e.g. [56], [57]), this is not always the case [32]. Therefore, it is entirely conceivable that in some species, the more tame, lower activity fish are preferentially harvested (e.g. [28]), whereas in others, the more active, bold ones face higher mortalities under fished conditions (e.g., [21]). However, because low activity fish tend also to be the more tame and less aggressive behavioural types [32], selection against high activity might often result in a corresponding decline in average boldness and aggression within a population of fish, which would in turn affect catchability negatively [18]. Most of these behavioural traits are also negatively correlated with the productivity of individuals such that fishery-induced selection acting on behaviour might also indirectly change life-histories and productivities. In fact, a recent survey of empirical studies [58] indicated that boldness, activity and/or aggressiveness are all positively related to food intake rates, productivity and other life-history traits such as growth. Thus, decreasing the number of individuals with vulnerable behavioural traits might ultimately have a negative effect on the population’s productivity [25].

One of the limitations of our study is that we assumed fishing styles rather than deriving them from observations of how fishers move. Although we believe that we have captured the most important styles of fishers, it would be preferable to link the fish movement data to realistic fishing patterns. We would therefore like to extend our modelling study using revealed behaviours of fishing vessels, such as those revealed by vessel monitoring systems (e.g. [35]). However, currently, it is rarely mandatory to use these type of devices in some of the fisheries we are mostly interested in (i.e., artisanal or recreational fisheries), contributing to the challenge to document typical fishing styles in nature. Because of the continued challenges of jointly modelling both fish and fisher movements, the linking of human (as predators) and fish (as prey for humans) data to quantify the number of encounters remains an important avenue for future research. Because behavioural reactions to gear exposure by fish and the behavioural adaptation of humans to compensate for altered fish behavioural phenotypes jointly determine catch rates, studying the resulting “red queen” between humans and fish from a behavioural perspective is recommended as a suitable direction for the future. One prominent null hypothesis emerging from the current work is then that there should be consistent selection toward low activity phenotypes in many exploited fish populations, leaving behind phenotypes and potentially genotypes that are more difficult to capture, in turn challenging the use of catch rates and other fishery-dependent data to index fish abundance reliably in long time-series [58]. Further studies are needed to understand how behaviour changes in response to fishing and how fishers in turn adapt their behaviour to maintain viable fisheries. This interaction seems to be ongoing since fishing started many decades ago. In light of this, it is surprising how little we know about which behaviours render a fish vulnerable to capture and how vulnerability is affected by human behavioural responses. We hope our work has contributed to this open question.
